# *Shigella sonnei* Bacteremia Presenting with Profound Hepatic Dysfunction

**DOI:** 10.1155/2017/7293281

**Published:** 2017-02-23

**Authors:** Oluwaseun Shogbesan, Andrew Rettew, Bilal Shaikh, Abdullateef Abdulkareem, Anthony Donato

**Affiliations:** ^1^Department of Internal Medicine, Reading Health System, Spruce Street and 6th Avenue, West Reading, PA 19610, USA; ^2^Department of Hematology and Oncology, Banner University Medical Center, Tuscon, AZ, USA; ^3^St Francis Medical Center, Cape Girardeau, MO, USA

## Abstract

Worldwide, Shigellosis is a significant public health issue, associated with nearly one million deaths annually. About half a million cases of* Shigella* infection are reported annually in the United States.* Shigella* bacteremia is uncommon and generally seen in children and immunocompromised adults. We present a case of a* Shigella sonnei *bacteremia with marked hepatic derangement in a 27-year-old previously healthy homosexual male with history of Roux-en-Y gastric bypass, who presented to the emergency room with a 4-day history of loose watery stool, abdominal cramps, nausea and vomiting, and yellow skin of 2-day duration. He reports similar diarrhea illness in two close contacts in preceding days. On examination, he was fully oriented but dehydrated, icteric, and febrile. Laboratory data revealed WBC of 2200/*μ*L, elevated AST and ALT (201 IU/L, 73 IU/L resp.), normal alkaline phosphatase, elevated total and direct bilirubin of 8.2 mg/dL and 4.4 mg/dL, albumin of 3.2 g/dL, INR of 2.9, prothrombin time of 31.7, and platelet of 96,000/*μ*L. Workup for infectious, autoimmune and medication-induced hepatitis, Wilson's disease, and hemochromatosis was negative. Abdominal ultrasound and computed tomography of the abdomen showed hepatic steatosis and right-sided colitis. Stool and blood cultures were positive for* Shigella sonnei*. He was treated with ciprofloxacin with improvement in liver function. Follow-up blood test 4 months later was within normal limits.

## 1. Introduction

Worldwide,* Shigella *is a common food-borne bacterial cause of dysentery and rarely causes bacteremia in the immunocompromised host [1, 2]. Infection is more common in developing countries and areas with poor hygiene and contaminated water sources, but sexual transmission has been reported among men who have sex with men (MSM) [1, 3–6]. We report a case of* Shigella* bacteremia in a homosexual male complicated by hepatic dysfunction.

## 2. Case Report

A 27-year-old African American male with a history of morbid obesity status post-Roux-en-Y gastric bypass 9 years prior presented with painful nonbloody mucoid diarrhea, icterus, and dark urine of four-day duration. He described recent travel with family to a beach resort in the United States during which he noted that two family members had developed a self-limited diarrheal illness. Three days after exposure, he noted abdominal cramps followed by hourly watery yellow stools without blood. He also had intermittent nausea and an episode of bilious vomiting and reports not eating since onset of diarrheal illness. He noticed a 17-pound weight loss over 1 week. He had no fevers, chills, or rash. There was no preceding history of eating uncooked fish or meats and he had no tick exposure. Medication history included Lisinopril and Amlodipine, and he denied any new medications or alcohol use. He admitted to homosexual intercourse.

He was acutely ill-looking with icteric sclera and dry mucus membranes. Vitals signs showed low-grade fever of 100.2°F and pulse rate of 126 beats per minute. Cardiac, lung, and skin examinations were unremarkable. Abdomen was diffusely tender with hyperactive bowel sounds without peritoneal signs.

Laboratory data was remarkable for leukopenia of 2200/*μ*L, hemoglobin of 12.7 g/dL, normal platelet of 158,000/*μ*L, sodium of 128 meq/L and potassium of 3.2 meq/L, acute kidney injury with creatinine of 1.59 mg/dL (baseline of 0.63 mg/dL), BUN of 41 mg/dL, and anion gap of 19. After intravenous hydration, hemoglobin dropped to 10.1 g/dL and platelet to 96,000/*μ*L. Sodium, BUN, and creatinine also improved with rehydration. He was also noted to have elevated transaminases AST 201 IU/L and ALT 73 IU/L, elevated total bilirubin of 8.2 mg/dL, direct bilirubin of 4.4 mg/dL, normal alkaline phosphatase of 66 IU/L, albumin of 3.2 g/dL, normal lipase of 40 U/L, and elevated lactate of 3.5 meq/L. INR was elevated to 1.8; however, he had normal haptoglobin 236 mg/dL (36–195 mg/dL) and LDH 177 IU/L (normal: 94–202 IU/L). Hepatitis panel was negative. Patient also tested negative for HIV, CMV, EBV, and Parvovirus B19 infection. Tylenol level was normal. Urinalysis showed moderate bilirubinuria. Stool study was positive for* Shigella sonnei* but was otherwise negative for bacteria, ova, or parasites.* Rickettsia rickettsii* IgM was positive and he was treated with Doxycycline. Other workup for tick-borne infection including Lyme,* Babesia*,* Ehrlichia*, and* Anaplasma* was negative. Autoimmune workups including ANA, antismooth muscle, liver/kidney microsome, and mitochondrial antibodies were negative. Alpha-1 antitrypsin and ceruloplasmin were also normal.

Abdominal ultrasound showed a diffusely increased heterogeneous liver echogenicity with underlying hepatomegaly consistent with hepatic steatosis. Hepatoportal duplex showed patent portal vein and hepatic artery. Computed tomography of the abdomen and pelvis was significant for hepatic steatosis, hepatomegaly, mild right colitis, and absence of gallstones. Magnetic resonance cholangiopancreatography (MRCP) was unremarkable with normal intra- and extrahepatic biliary tree.

During his hospital course, bilirubin trended up to 9 mg/dL and INR peaked at 2.9. Patient was not encephalopathic. Given impending hepatic failure, recommendation was made to transfer the patient to a transplant center but he declined. He was treated with N-acetylcysteine. Blood culture initially reported as Gram-negative rods presumptively* Escherichia coli (E. coli)* was later confirmed to be* Shigella sonnei*. Urine culture remained negative. He was treated with ciprofloxacin based on sensitivity results with an improvement in diarrhea frequency and downward trend in transaminases. He remained stable and was discharged home on Day 8. He was doing well 4 months later with normal cell counts, normal liver, and kidney function.

## 3. Discussion

*Shigella* has a low infectious dose, requiring as low as 10 organisms for infection, and it is easily transmitted from person to person [20]. Outbreaks are common in day care centers, institutions, and urban areas with crowded living conditions [6]. In the United States and other developed countries, transmission is usually fecal-oral from symptomatic patient and fecal contamination of raw vegetable has been identified in outbreaks [3, 6]. Average incubation period is three days and patients typically presents with small volume watery to mucoid diarrhea, abdominal pain, vomiting, fever, and bloody diarrhea.

*Shigella sonnei* is responsible for three-quarters of all cases in the US, while* Shigella flexneri* is predominant in developing countries [1, 21, 22]. Complications range from rare intestinal complications of toxic megacolon, intestinal obstruction to systemic complications of hypovolemia, seizures, hyponatremia, and leukemoid reaction.

This case is unique for several reasons, first because* Shigella* bacteremia is uncommon in adults and even less common in the absence of underlying immunosuppression [23, 24]. Our patient was not immunocompromised and extensive workup to detect underlying predisposition was negative. Patient did have a history of Roux-en-Y gastric bypass and possibility of underlying nutritional deficiency cannot be conclusively excluded. Morduchowicz et al. reported 27 cases of* Shigella* bacteremia of which 16 had an underlying predisposing condition [12]. Appannanavar et al. also reported a case of bacteremia in a renal transplant patient and two other cases in postrenal transplant are reported in literature [8, 25, 26]. Hawkins et al. presented a total of 9 cases of* Shigella *bacteremia in adults with underlying predisposing comorbidities including the use of immunosuppressant, diabetes mellitus, alcohol liver disease, HIV/AIDS, and malignancy [16]. Similar to our case, Huynh et al. presented an otherwise healthy male without identified comorbidities; he was a MSM [19].

Increasing cases of Shigellosis have been reported among MSM [4, 19]. In a case series of 466 cases of* Shigella* bacteremia, two-thirds of reported cases in adult males were in MSM [19]. Outbreak of* Shigella *among MSM has also been reported in Europe, USA, and Australia [27–31]. There is no increase in incidence of* Shigella* bacteremia among HIV infected individuals, although increased mortality has been noted in HIV-positive patients with* Shigella* bacteremia [1].

Additionally, to our knowledge, this is the first reported case of hepatic manifestation of Shigellosis in an adult. Our patient had coagulopathy with bilirubinemia, elevated transaminases, and thrombocytopenia but was not encephalopathic and thus not by definition in fulminant liver failure. Workup for viral hepatitis, toxin or drug hepatitis, autoimmune hepatitis, Wilson's disease, malignant, or vascular etiology was negative in the setting of* Shigella* bacteremia. His underlying fatty liver disease might have played a role. Despite the absence of encephalopathy, our patient was considered for referral to transplant center given rising bilirubin and INR concerning for impending fulminant hepatic failure. Guided by evidence of increased transplant free survival in hepatic failure, he was treated with N-acetylcysteine when he declined transfer. Of note, liver function improved with treatment of* Shigella* infection. A case of fulminant hepatic failure in a three-and-a-half-year-old boy with* Shigella* bacteremia also made clinical and biochemical improvement with Shigellosis treatment [32].

Our patient tested positive for* Rickettsia rickettsii* IgM and this was considered to be a false positive result in the absence of prominent fever and classical rash which is seen in 90% of cases. Also our patient did not have other common features of Rocky Mountain spotted fever (RMSF) including headaches, myalgia, and arthralgia and no preceding tick exposure. In advance cases of RMSF, elevations in serum aminotransferases and bilirubin and prolongation of prothrombin time are possible; it was however considered an unlikely explanation in our index case. False positive* Rickettsia* IgM is known to occur in presence of other bacteria pathogen due to cross reactivity to similar lipopolysaccharide [33, 34].

Huynh et al. reported a case of* Shigella* bacteremia in 34-year-old MSM in which a positive blood culture was initially reported as* E. coli* [19].* E. coli* and* Shigella* both Enterobacteriaceae are genomically similar with identical O- antigens and similar virulence determinants [35, 36]. Rapid testing methods such as the natrix-assisted laser desorption ionization-time of flight mass spectrometry (MALDI-TOF MS) used in many diagnostic microbiology laboratories cannot distinguish between both [37, 38]. In patients with discordant* Shigella* stool culture and* E. coli* bacteremia, conventional and serologic testing is required to correctly identify* Shigella* and prevent an unnecessary search for an additional infection source.

We found 18 reported adult cases of* Shigella sonnei* bacteremia in literature. 14 of the 18 patients had an underlying immunocompromising comorbidity including diabetes mellitus, AIDS, malignancy, and postorgan transplantation (Table 1). One of the 4 patients without an identified comorbidity was a MSM similar to our case. All 3 patients with underlying solid organ malignancy died during the course of* Shigella* bacteremia. Over all, there were 6 deaths, 5 of which had significant underlying comorbidities.

Shigellosis is often self-limited and infection tends to clear spontaneously in most individuals. In severe cases with complications, including bacteremia, treatment is indicated. First-line treatment is with fluoroquinolones. However, given increasing antibiotic resistance, particularly strains acquired from Asia and Africa, susceptibility testing is important [3, 39, 40]. Of note, reduced susceptibility and/or resistance to ciprofloxacin has been reported in the United States [21].

## Figures and Tables

**Table 1:** Reported cases of adult *Shigella sonnei* bacteremia.

Author	Comorbidity	Outcome
Winter and Harding 1962 [7]	None	Recovery
Netter et al., 1974 [8]	Renal transplant	Recovery
O'Connor and O'Callaghan 1981 [9]	Marrow Aplasia	Death
Roncoroni et al., 1984 [10]	(1) CKD	Recovery
	(2) None	Death
Alkan et al., 1985 [11]	Metastatic adenocarcinoma	Death
Morduchowicz et al., 1987 [12]	(1) None	Recovery
	(2) Diabetes mellitus	Recovery
Dronda et al., 1988 [2]	Diabetes mellitus	Recovery
Christensen et al., 1990 [13]	Congenital antithrombin III deficiency, postsplenectomy	Recovery
Seymour et al., 1994 [14]	(1) AIDS	Death
	(2) Alcoholic liver disease	Recovery
Kenet et al., 1994 [15]	Metastatic breast cancer	Death
Hawkins et al., 2007 [16]	(1) Multiple myeloma	Recovery
	(2) Diabetes mellitus	Recovery
Liu et al., 2009 [17]	Lung cancer	Death
Markham et al., 2012 [18]	AIDS	Recovery
Huynh et al., 2015 [19]	None	Recovery
